# Supplementary material for: Reconciling Mining with the Conservation of Cave Biodiversity: A Quantitative Baseline to Help Establish Conservation Priorities
Source: PLoS One. 2016 Dec 20;11(12):e0168348. doi: 10.1371/journal.pone.0168348 (PMC5173368; doi:10.1371/journal.pone.0168348)
Supplement: S1 Dataset — (ZIP) [file pone.0168348.s002.zip › Taxa/Serra Norte/SN_2013/PES/Lista PESL-03.pdf]

CAVIDADE PESL-0003

Classe	Ordem	Fam/Outros	Gên/Outros	Espécie	S-U	U-S	Zon.
Arachnida	Acari	Macronyssidae		sp.1		X	E
Arachnida	Acari	Rhagidiidae		sp.1		X	P
Arachnida	Araneae	Corinnidae		jovem	1		P
Arachnida	Araneae	Mysmenidae	*Microdipoena*	sp.1	X		P
Arachnida	Araneae	Pholcidae	*Mesabolivar*	*eberhard*	X	X	E/P
Arachnida	Araneae	Salticidae		sp.2		X	P
Arachnida	Araneae	Theraphosidae		jovem		6	E
Arachnida	Araneae	Theridiidae	*Achaearanea*	sp.1		X	P
Arachnida	Araneae	Theridiosomatidae	*Ogulnius*	sp.1	X	X	P
Arachnida	Opiliones	Escadabiidae		sp.2		X	P
Arachnida	Opiliones	Sclerosomatidae	*Prionostema*	sp.1	X	X	P
Arachnida	Palpigradi	Eukoeneniidae	*Leptokoenenia*	*thalassophobica*		X	P
Arachnida	Pseudoscorpiones	Chernetidae	*Spelaeochernes*	sp.1	X	X	E/P
Entognatha	Diplura	Japygidae		sp.1		X	P
Insecta	Blattodea	Polyphagidae		jovem	2	1	E
Insecta	Coleoptera	Carabidae		jovem	X		P
Insecta	Coleoptera	Scarabaeidae		jovem		X	P
Insecta	Coleoptera	Tenebrionidae		jovem		X	P
Insecta	Diptera	Chloropidae		sp.	X		E
Insecta	Diptera	Dolichopodidae		sp.	X		E
Insecta	Diptera	Psychodidae	*Sciopemyia*	*sordellii*	X	X	P
Insecta	Diptera	Tipulidae	Tipulinae	sp.	X		E/P
Insecta	Heteroptera	Reduviidae	Emesinae	sp.2	1		E
Insecta	Heteroptera	Reduviidae	Reduviinae	jovem	2	3	E/P
Insecta	Homoptera	Cicadellidae		sp.4	X		E
Insecta	Homoptera	Cixiidae		jovem	X	X	E/P
Insecta	Hymenoptera	Formicidae	*Camponotus*	sp.1	X	X	E/P
Insecta	Hymenoptera	Formicidae	*Crematogaster*	sp.3		X	E
Insecta	Isoptera	Termitidae	*Nasutitermes*	sp.	X	X	E
Insecta	Lepidoptera	Noctuoidea		sp.12	3		P
Insecta	Neuroptera	Myrmeleontidae		jovem	2	2	E/P
Insecta	Orthoptera	Phalangopsidae	*Paraclodes*	sp.	2	1	E
Insecta	Orthoptera	Phalangopsidae	*Phalangopsis*	jovem	30	1	P
Insecta	Psocoptera	Dolabellopsocidae	*Dolabellopsocus*	sp.1		X	E
Insecta	Psocoptera	Epipsocidae	*Epipsocus*	sp.1		X	E/P
Malacostraca	Isopoda	Armadillidae		sp.1	X		P
Amphibia	Anura	Strabomantidae	*Pristimantis*	cf. *fenestratus*	3		P
Mammalia	Chiroptera	Emballonuridae	*Peropteryx*	sp.	4	3	P
Mammalia	Chiroptera	Furipteridae	*Furipterus*	*horrens*		3	P
